# Retrosternal Goiter: A couple of classification methods with computed tomograpy findings

**DOI:** 10.12669/pjms.346.15932

**Published:** 2018

**Authors:** Gokhan Perincek, Sema Avci, Pinar Celtikci

**Affiliations:** 1 *Dr. Gokhan Perincek, Department of Pulmonology, Kars Harakani State Hospital, Kars, Turkey*; 2 *Dr. Sema Avci, Department of Emergency Medicine, Sabuncuoglu Serefeddin Research and Training Hospital, Amasya, Turkey*; 3 *Dr. Pinar Celtikci, Department of Radiology, Kars Harakani State Hospital, Kars, Turkey.*

**Keywords:** Retrosternal, Goiter, Anatomical, Tomography, Classification

## Abstract

**Objectives:**

The retrosternal goiter (RSG), which can be defined by different classification and its incidence between 2% and 26% of all thyroidectomized patients, is a thyroid gland disease. Our aim was to classify RSG cases with a couple of different ways, which we have detected in computed tomography (CT) imaging of the thorax.

**Methods:**

In this retrospective study conducted at Kars Harakani State Hospital Pulmonary Medicine Polyclinic between June 2014 and June 2017 in which 176 patients were included. The age, sex, diagnostic codes, retrosternal extension of the thyroid gland (aortic upper arch, aortic reaching arch and aortic inferior arch), extension type (prevascular, paratracheal retrovascular and retrotracheal), extension amount (mm) (<50% and 50%<) of thyroid gland of the patients were assessed.

**Results:**

About 56.25% (n=99) were female and the mean age was 65.9±11.4 years. The most common co-morbid disease in patients with RSG was Chronic Obstructive Pulmonary Disease (COPD) (52.3%). Thirty nine (22.2%) of the patients had associated nodule, 16 (9.1%) had accompanying tracheal pressure and one patient had nodule and tracheal pressure. 27.3% of the patients’ gland’s right lobe and 28.9% of the patients’ left lobe were extended >50% below the thoracic entry. Left thyroid gland’s (90.3%) retrosternal extension and aortic arch spread (91.2%) were more. When classified according to the trachea, the left lobe’s paratracheal and retrovascular extension (50.9%) was more. Extension amounts were similar for both thyroid lobes.

**Conclusion:**

In patients who have retrosternal goiter, goiter spread can be defined with multiple classification with thorax CT.

## INTRODUCTION

The concept of “goiter” originating from the Latin word “tumidum gutter” means that thyroid gland grows up to twice its size, or it is heavier than 40 grams.^1^ Goiter is a thyroid gland disease that affects about 5% of the world.^2^

An enlarged thyroid gland most often causes subclinical hypothyroidism. Increased level of thyroid stimulating hormone (TSH), iodine deficiency in the diet and clinical reflection of many pathologies may cause this.^1^ Though 85-90% of the goiters are in the cervical region and 10-15% in the intrathoracic region, although they differ between the studies.^3^ The concept of substernal goiter was first used by Haller and since then this concept has been controversial and it still does not have a single definition today.^4^,^5^ Retrosternal, substernal, intrathoracic, or mediastinal goiter concepts are the concepts that are used for goiters that extend downwards from the thoracic entry, and that do not currently have an agreement about them.^1^

The incidence of retrosternal goiter (RSG) varies between 2% and 26% of all thyroidectomized patients.^5^ One of the commonly accepted RSG definitions is that the gland extends downward from the thoracic patency.^5^

The rate of mortality and morbidity is very low with surgery performed in the treatment of patients with retrosternal goiter.^4^ Although there is treatment of RSG with thyroid hormone therapy or radioactive iodine ablation therapy other than surgery, they are not successful as surgery.^6^

Patients with retrosternal goiter may have symptoms because of pressure on the esophagus, nerve and vascular structures, airway pressure symptoms such as dyspnea, orthopnea and stridor, and less frequent superior vena cava syndrome.^6^ Patients may present with complaints of dysphagia and hoarseness, and patients may detect hyperthyroidism or metabolic findings of thyrotoxicosis.^6^ Approximately 5-40% of patients are asymptomatic and are detected randomly by chest radiography or thorax CT.^6^ CT imaging of the neck and chest region is the best examination that can scan and identify intrathoracic goiter components.^5^ Our aim in this study was to determine the frequency of RSG and classify the CT findings of patients with RSG by several different methods.

## METHODS

For this study; with the approval of the Kafkas University Ethics Committee, we retrospectively analyzed 4334 thoracic CT images, with or without contrast, of patients with Kars Harakani State Hospital Pulmonary Medicine Polyclinic between June 2014 and June 2017. One hundred eighty cases with retrosternal thyroid gland were reevaluated and typed. Four cases were excluded from the study (two cases were excluded because of artifacts, one case because of a mass which is destructive to the sternum, one case because of the thoracic wall and the spinal deformity, all of which were obstacles to measurement). The remaining 176 patients constituted the study group. The manubrium stern upper end was taken as a reference for retrosternal thyroid gland measurement. The age, sex, diagnostic codes, retrosternal extension of the thyroid gland (aortic upper arch, aortic reaching arch and aortic inferior arch), extension type (prevascular, paratracheal retrovascular and retrotracheal), extension amount (mm) (<50% and 50%<) of patients were assessed.

The data was analyzed with SPSS for Windows version 23.0. Mean, standard deviation in descriptive statistics of continuous variables; were expressed categorical variables in numbers and percentages.

## RESULTS

Of the 176 patients included in the study, 43.75% (n=77) were male, 56.25% (n=99) were female and the mean age was 65.9±11.4, 59.6% (n=59) of females and 45.45% (n=35) of males were over 65 years old. The most common co-morbity for patients with retrospective goiter was Chronic Obstructive Pulmonary Disease (COPD) (52.3%). 39 (22.2%) of the patients had associated nodule, 16 (9.1%) had accompanying tracheal pressure and one patient (0.6%) had nodule and tracheal pressure (Table-I). We detected RSG in 4.15% of the thorax CT images.

**Table-I:** The diagnostic need for computed thorax CT of the patients with RSG.

	n (%)
***Diagnosis***	
COPD	92 (52.3%)
Asthma	45 (25.6%)
Dyspnea	7 (4%)
Hemoptysis	3 (1.7%)
Sarkoidosis	1 (0.6%)
Pulmonary thromboembolic	2 (1.1%)
Chest pain	4 (2.3%)
Pneumonia	4 (2.3%)
Tuberculosis	1 (0.6%)
Routine chest radiography	2 (1.1%)
Cough	4 (2.3%)
Trauma	1 (0.6%)
Lung cancer	1 (0.6%)
Gastro-oesophageal reflux	1 (0.6%)
Bronchitis	1 (0.6%)
Myalgia	7 (4%)

Anatomical classification was made with thorax CT findings of the patients. 27.3% of the patients’ gland’s right lobe and 28.9% of the patients’ left lobe were extended more than 50% of their sizes and reaching below the thoracic entry. Left thyroid gland’s (90.3%) retrosternal extension and aortic arch spread (91.2%) were more. When classified according to the trachea, the left lobe’s paratracheal and retrovascular extension (50.9%) was more. Extension amounts were similar in terms of mm for both thyroid lobes (Table-II).

**Table-II:** CT findings of RSG spread.

	Right lobe	Left lobe

	ort±ss / n(%)	
Retrosternal extension	110 (62.5%)	159 (90.3%)
***Extension location***		
Aortic upper arch	100 (90.9%)	145 (91.2%)
Aortic reaching arch	10 (9.1%)	13 (8.2%)
Aortic inferior arch	-	1 (0.6%)
***Extension type***		
Prevascular	51 (46.4%)	75 (47.2%)
Paratracheal, retrovascular	57 (51.8%)	81 (50.9%)
Retrotracheal	2 (1.8%)	3 (1.9%)
Extension amount	19.31±7.94	19.8±10.06
***>%50 extension***		
<%50	80 (72.7%)	113 (71.1%)
>%50	30 (27.3%)	46 (28.9%)

## DISCUSSION

Approximately 85-90% of the enlarged thyroid glands appear as a mass which can press in the cervical region and approximately 10-15% of them appear as a mass which can press in the retrosternal region.^3^ Together with the incomprehensibility about goiter still, the incidence of RSG differs between studies. In a national study conducted in the United States; it was reported that the average age of the patients was 57.78, hypertension was the most frequent complication of RSG, and is seen more frequently in women. In this study, the incidence of RSG in the United States in the whole population was 4.98%.^7^ In another study conducted in the United States, the incidence of RSG was 6.9%, the average age of the patients was 57.8 and the ratio of female to male was 3:1.^8^ In studies in our country that patient numbers are lower; in the study that conducted by Atalay et al. the incidence of RSG was 8.1%, the mean age was 62.87 and 54.1% of the patients were male.^9^ In another study; Yazıcıoglu et al. determined that 61.9% of patients with RSG were female and the mean age was 56.4 years.^4^

RSG, which shows differences in incidence, gender distribution and age, is more common in women in studies in the United States and differs in gender in our country. In the national study conducted in the United States, the most common comorbidity in the studies is hypertension, and in our study, the most common comorbidities are COPD and asthma, and this can be only due to the fact that we evaluated patients referred to the Pulmonary Medicine Policlinic. Although Chronic Obstructive Pulmonary Disease or Asthma were the most common comorbidities, radiological tracheal compression findings were present in only 9.7% of the patients.

Although there are many classification methods for RSG nowadays, the most commonly used classification is that the size of the thyroid gland below the thoracic entry is more than 50%.^10^ Rafaelli et al. about the classifications for RSG; have defined it as having more than 50% of the thyroid gland or any thyroid nodule in the mediastinum while the patient was lying on the operating table before the thyroidectomy.^11^ In our study, more than 50% of the right lobe was in the thoracic entry in the 27.3% of the patients and more than 50% of the left lobe is in the thoracic entry in the 28.9% of the patients.

Patients with retrosternal goiter radiological findings such as trachea deviations can be seen as well as compressive symptoms such as cough, dyspnea, stridor and dysphagia.^12^ Tracheal pressure of massive goiters detected in thyroidectomy patients, which constitute a large proportion of surgical patients, is not uncommon.^13^ In the thyroidectomy series of Sajid et al., 2.68% of patients had clinical and radiological findings of tracheal pressure, and 84% of all patients had radiological evidence of tracheal compression.^13^ The correlation between computed tomography retrosternal spread or size of the goiter with trachea deviations and the presence of symptoms may be weak.^12^ Radiologic evaluation of our patients showed that 22.2% had nodule, 9.1% had tracheal pressure, and in only one patient two of them were together. In the study of Atalay et al., 25% of the cases had tracheal pressure and 70.8% had nodules.^9^ Rugiu et al. in their RSG study, the rate of tracheal deviation and pressure detection on CT imaging was 78%.^14^

Computed tomography is the gold standard in the RSG classification and is classified according to sagittal (cranio-caudal), axial (anteroposterior) and coronal (latero-lateral) planes.^1^,^15^ In our study, the RSG was classified according to the aortic arch, according to the trachea and the extent of more than 50% of the gland.

To assess the spread of mediastinal goiter, Benbakh et al. classified 90% of patients according to the aortic arch and trachea in CT and they did not separate the thyroid lobes, as right and left, differently from ours. In this study, prevascular and aortic upper arch were the most common type of dissemination.^16^ According to the anatomical classification of Huins et al. which is made before surgical approach by Dempsey et al., prior to the surgical approach, the spread of RSG above the aortic arch was the most common group.^17^ According to retrosternal goiter scan CT images of Sakkary et al. the most common spread types were the vertical distribution of the left lobe aortic upper arch and also longitudinally pretracheal spread.^5^ Left lobe spread to aortic upper arch was the most common radiological finding in our patients too. The most common RSG spread according to Mercante et al.’s Cross-Sectional Imaging CT classification system was in between the thoracic inlet and aortic arch convexity as cranio-caudal.^15^ In the study of Khairy et al., the gland’s left lobe extension was more similar to ours.^18^ On the contrary, in the study of Rugiu et al., the right lobe retrosternal spread was greater.^14^ As a different classification example, in the study of Malvemyr et al. 33.3% of the patients with RSG had the most prevalence between the thoracic entry and the aortic arch convection as prevascular and cranio-caudal anteroposterior.^19^ In our study, the left lobe’s paratracheal and retrovascular spread were greater.

Our study in which a large number of CT images were evaluated can give accurate information regarding the frequency of RSG in our country, age and gender. Inclusion of only patients who were referred to the Pulmonary Disease Policlinic provides limited information on the detection of retrosternal goiter associated diseases. As a result, it is possible to define the distribution of goiter by performing more than one classification in patients with retrosternal goiter with thorax CT which is the gold standard in the diagnosis.

### Author’s Contribution

**GP** did data collection, conceived and designed this study.

**SA** manuscript writing, statistical analysis and final approval of manuscript.

**PC** data collection, data interpretation and editing manuscript.
